# Enhanced photoconductivity in CdS/betanin composite nanostructures†

**DOI:** 10.1039/c7ra13116j

**Published:** 2018-03-21

**Authors:** N. Susha, K. Nandakumar, Swapna S. Nair

**Affiliations:** Dept. of Physics, Central University of Kerala Kasaragod India – 671314; International and Inter University Centre for Nanoscience and Nanotechnology, Mahatma Gandhi University Kottayam India – 686 560 swapna@cukerala.ac.in

## Abstract

Development of novel materials for thin film solar cells are gaining significant attention due to their tunable wide bandgap and extensive application potential in flexible energy harvesters. CdS is a known window material for thin film solar cells. Tuning of the photoconductivity of CdS by doping, substitution and grain size tailoring is widely attempted by researchers. Inorganic core/shell structures like CdS/CdSe, CdS/ZnS *etc.* are other possible candidates with band gap tailorability. However, such attempts are rare in tailoring the photoconductivity by providing an organic shell over the inorganic core. Here the authors synthesised CdS/betanin core/shell structures using wet chemical routes. Spectroscopic studies show that the composite structure is core/shell like, with CdS as the core and betanin (a natural dye), as the outer shell with an average core particle size of 10 nm. The absorption spectra of the composite system show the signature of an additional band in the lower wavelength region and it is redshifted with increase in betanin percentage. The intermediate band observed in the energy of ∼1.75 eV, helps CdS to enhance the rate of absorption. Simultaneous absorbance of lower and higher energy photons from the solar radiation can increase the efficiency of CdS based solar cells. A huge enhancement in conductivity is observed in CdS/betanin composites on illumination with white light due to the transfer of photogenerated electrons from the conduction band of betanin dye to the conduction band of CdS.

## Abstract 2

The synthesis is described of inorganic/organic hybrid nanostructured composites based on CdS/betanin with enhanced photoconductivity due to the transfer of photogenerated electrons from the conduction band of betanin to the conduction band of CdS.

## Graphical abstract

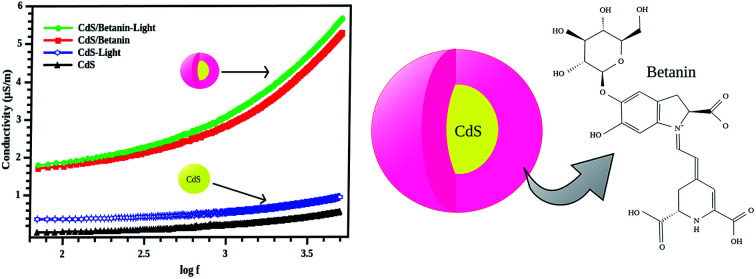

## Introduction

Semiconductors, especially silicon (Si) based electronic devices, inexorably paved the way for the modern technology of computation and electronics. The innovation and tailoring of “nano” introduced wide varieties of material choices for the device fabrication other than silicon, which has had a dominance in the semiconductor industry for the last 50 years. Exploring an efficient low-cost alternative for Si, to meet its marketing demands, is still a challenge for the researchers working in the field of electronic device fabrication. Alterations in electronic gadgets such as scaling down, have extended the life span, while higher efficiency empowered their advancement in an unlimited number of applications and hence researchers are so energized in embracing wide band gap materials in electronic device fabrication.^1,2^ Wide bandgap semiconductors (materials which have band gap relatively greater than conventional silicon) like silicon carbide (SiC, band gap 3.3 eV) and gallium nitride (GaN, band gap 3.4 eV) thus emerged as the front running solutions for the power and heat issues of silicon (band gap 1.1 eV). Comparing to Si, SiC is the most maturely developed wide band gap material which has high-temperature/voltage stability with 20% higher efficiency.^3,4^ But presently its production is much more expensive than silicon. GaN is one among the widely explored cost effective alternative that offers similar performance of SiC. However, the problems associated with the synthesis of GaN demands alternative material search in this regard.

It is reported^5^ that tailoring the size, structure, morphology and chemical composition of nanomaterials can deliberately tune their properties and its integration leads to nanoscale device fabrication. So, the migration to novel materials other than conventional SiC and GaN can pave way to enhanced efficiency of electronic devices.

Quantum dots, the ultrafine nanoparticles (size below 10 nm), offer higher band gap than their bulk cousins due to the size dependent quantum confinement effects. This property of quantum dot helps them to harvest the hot electrons from the material with high energy photons and generating multiple charge carriers which offers new ways to attain greater efficiency in the next generation solar cells.^6^ For the last few years, II–VI group semiconductors attracted researchers owing to its wide band gap, availability, easy synthesis and band gap tunability.^7–9^ Among them, CdS is one of the most prominent candidate which has potential application in the field of optoelectronics. Tailoring of band gap of CdS is possible by varying the experimental conditions such as concentration, pH, stoichiometry, temperature *etc.* Experimentalists used organic as well as inorganic surfactants^10–13^ in conjunction with the precursors to stabilize the particle growth and to avoid agglomeration in CdS nanoparticles. Accordingly, a large fraction of the particles is observed to be distributed in the size range of 2–10 nm (^ref. 10^) and are categorized as semiconductor quantum dots (SQDs).

In solar cells, semiconductor has to fulfil both the task of light absorber and charge carrier transporter. But in Dye Sensitized Solar Cells (DSSC), light absorption is the responsibility of “sensitizer”, and the semiconductor in which the sensitizer is attached transports the charge.^14^ Researchers tried both organic and natural dyes as sensitizer and studied how effectively it absorbs the light. TiO_2_ is a well-known charge transporter which has wide band gap, later researchers replaced it by other wide band gap oxides such as ZnO,^15^ Nb_2_O_5_, SnO_2_, In_2_O_3_, WO_3_, Ta_2_O_5_, ZrO_2_,^16^*etc.* Researchers also examined the performance of II–VI group elements such as CdSe, CdTe, and CdS semiconductors as a central part of DSSC.^17^

**Fig. 1:**
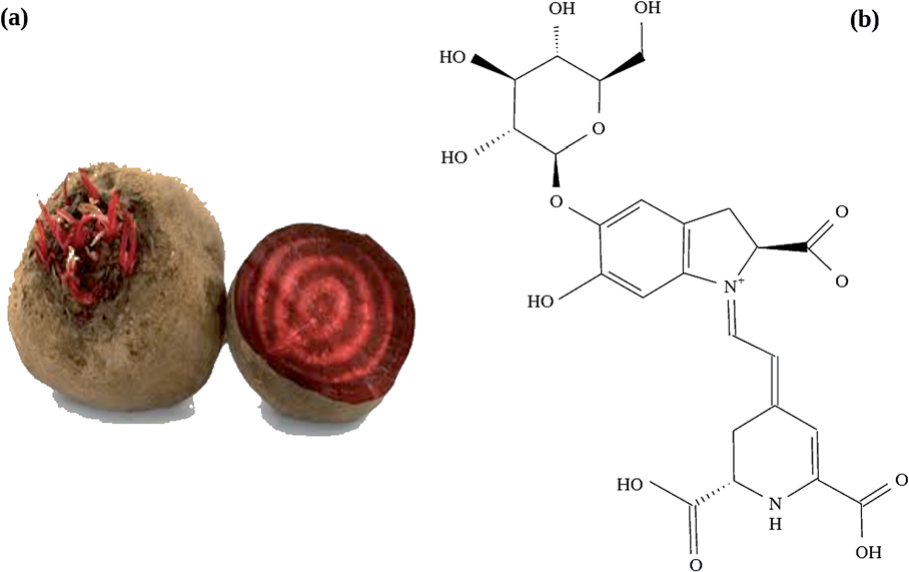
(a) Edible vegetable beet root and its inner portion, from which the betanin dye solution is extracted out using boiling method and (b) the structure of betanin with two nitrogen bonds –NH– and =N– with molecular formula C_24_H_27_N_2_O_13_.

Wide varieties of synthetic as well as natural dyes are already being a part of DSSC research. In addition to DSSC, dyes are used in core/shell nanostructure as both core and shell depending on the application of interest. Dye based organic/inorganic core/shell structure has tremendous application in the field of material science, microelectronics and biotechnology. Here, in the present investigation, we have used a natural dye (betanin, C_24_H_27_N_2_O_13_) extracted from the red beetroot (*Beta vulgaris*) (Fig. 1) – a well-known antioxidant^18,19^ as a medium for the growth of CdS nanoparticles. CdS and the CdS/betanin composite nanostructures are then structurally characterized for crystallinity and phase purity and optically characterized for absorption and photoconductivity.

## Experimental

### Synthesis

Chemical co-precipitation method is opted for the synthesis of CdS nanoparticles with CdSO_4_, CS(NH_2_)_2_ and NH_4_OH solutions as cadmium source, sulfur source and complexing agent respectively. To the CdSO_4_ solution, NH_4_OH is added dropwise until the pH value reaches 10. After 15 minutes, the precipitate is filtered and dried in air. The betanin solution is prepared from the fresh beet root using deionized water as a solvent. No acidification^20^ is tried here. Dark pink coloured solution obtained is kept in a conical flask. The procedure of CdS synthesis is repeated with different betanin amount (6%, 10%, 20% and 50%). The obtained precipitate is filtered, dried and grinded well. The colour of the sample varies from light yellow (without betanin) to dark brown as the dye content increases (Fig. 9).

### Characterisation

Structural characterisation of the samples is done using X-ray diffraction (XRD Rigaku Miniflex 600 with CuK_α_ = 1.5406 Å) and the patterns are recorded in the *θ*–2*θ* mode for the range 20–60°. Thermal analysis of the samples is done using thermogravimetric analysis (TGA, PerkinElmer–STA 6000). Shape and size of the particles, which are the property deciding factors of nanoparticles, are recorded by high resolution Transmission Electron Microscopy (TEM, JEOL, JEM-2100). The surface chemistry of the sample is done by X-ray Photoelectron Spectroscopy (XPS, PHI 5000 Versa Probe II, ULVAC PHI-USA) with Al-Kα source (power 33 W). PerkinElmer (Lambda 35) UV-Vis spectrophotometer is used to record its wavelength (380–800 nm) depended absorption spectrum. Precision impedance analyser (Wayne Kerr, 6500 B) is used to study the dielectric properties as well as the photoconductivity (using 300 watt incandescent bulb) of the samples.

## Results and discussion

### X-ray diffraction

**Fig. 2:**
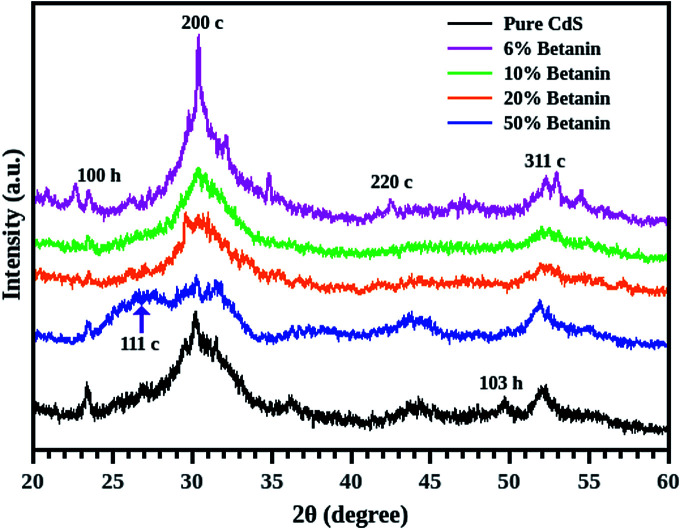
X-ray diffraction patterns of pure CdS and CdS/betanin composites (6% – magenta, 10% – green, 20% – red, 50% – blue) shows mixed crystal structure. Broadening of the peak is observed in the composite system with increase in dye content.

Diffraction patterns of pure CdS as well as CdS/betanin composite are shown in Fig. 2. Generally, CdS exists in two crystal forms – hexagonal and cubic, of which the hexagonal form is more stable^21^ at room temperature. From the figure, it is clear that the CdS exhibits mixed crystal structure with less intense hexagonal peak. As the betanin percentage increases, the peak corresponding to the plane [200 c] diminishes and become broad due to the smaller particle formation. This is because of the shelling of betanin dye. The grain growth dynamics may be rather slow than the shell formation^22^ resulting in small sized core CdS particles. Further grain growth is prevented because of the organic shell of betanin. The composite with 6% dye content has impurity peaks between 30° and 35° due to the unreacted cadmium compounds. This may be resulted from the slow reaction rate induced by the betanin dye. For the sample containing 50% of betanin, the highest electron density is observed for the cubic plane of (111 c) which indicates a change in orientation for the CdS nanocrystals. The choice of reactants, synthesis techniques and conditions can influence the formation of CdS, and the possibility of mixed phase formation is quite natural in chemical synthesis. X-ray diffraction data showed that most of the formed nanocrystals stabilizes in the cubic phase.

### Thermogravimetric analysis

**Fig. 3:**
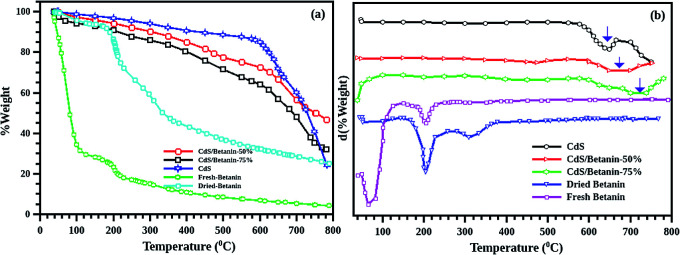
(a) TG curve of CdS (blue), fresh (green) and dried betanin (cyan), CdS/betanin composites with dye content 50 (red) and 75% (black) showing the percentage of weight loss during the heat treatment. Sudden loss of mass near 200 °C shows the loss of water content in both betanin. From the derivative TG curve (b), it is clear that pure CdS has stability upto 644 °C while the composite structure has higher thermal stability than CdS (upto 723 °C for the sample with 75% of dye).

The dynamic TGA (mass variation with temperature at constant heat rate) measurements are conducted on 5 samples including fresh betanin, dried betanin, CdS and CdS/betanin composites (50% and 75%) (Fig. 3). As the fresh betanin has larger water content, sudden loss of mass is observed in the temperature change of 40–100 °C. The degradation of the betanin dye is observed within the temp. of 204 °C. The dried beetroot has also similar behaviour except the huge mass change at the beginning of the experiment. In the case of pure CdS powders, thermal stability (16% loss of mass) is observed upto 644 °C. The 50% betanin added CdS showed thermal stability upto 673 °C while the 75% betanin CdS core system showed stability upto 723 °C. These results hint towards complete shelling of betanin on CdS nanoparticles (as no low temperature peaks corresponding to pure betanin is observed) the core/shell structure also ensured better stability with disintegration temperature.

### Transmission Electron Microscopy

**Fig. 4:**
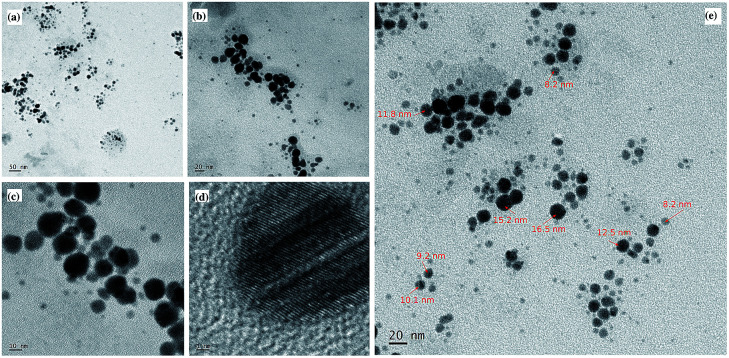
High resolution transmission electron micrograph of CdS/betanin composite (50%) nanoparticles (a–e). Most of the particles are spherical in shape and found in the size range of 8–17 nm with an average size of 10 nm.

Morphological analysis of CdS/betanin composite (50% betanin) is carried out by employing TEM and the results are shown in Fig. 4. Here, all the particles are found to be spherical in shape. No irregularity in shape is observed. All particles are found to be below 17 nm (average particle size is 10 nm) in diameter. Particle which has a radius less than the exciton Bohr radius limit of 2.9 nm (^ref. 23^) is considered as quantum mechanically confined particles. This confinement of particle leads to the formation of discrete energy levels. Here most of the particles are above 6 nm (8–17 nm) in diameter and expected to behave like nanoparticles with weak to negligible quantum confinement effects. Similar behaviour is obtained for Ag/dye composite nanoparticles, a theoretical and experimental study of inorganic/organic core/shell nanoparticle^24^ by Lebedev *et al.*^25^ According to them, the silver/dye system behaves like a core/shell nanoparticle by varying the size of the core. Since TEM images does not provide any evidence for the presence of dye, we can only assume that the CdS/betanin composite nanoparticle is behave like core/shell nanoparticles with CdS as core and betanin as the shell.

### X-ray photoelectron spectroscopy

The surface composition of the CdS/betanin composite is analysed by employing XPS (X-ray Photoelectron Spectroscopy or ESCA, Electron Spectroscopy for Chemical Analysis), a very sensitive technique for the chemical analysis. The characterisation is carried out on surface and depth profiling was done upto 10 nm. Here, the solid samples are irradiated with X-rays in vaccum and the emitted electron energy is analysing. Every component has distinctive set of binding energies and has unique spectrum.

**Fig. 5:**
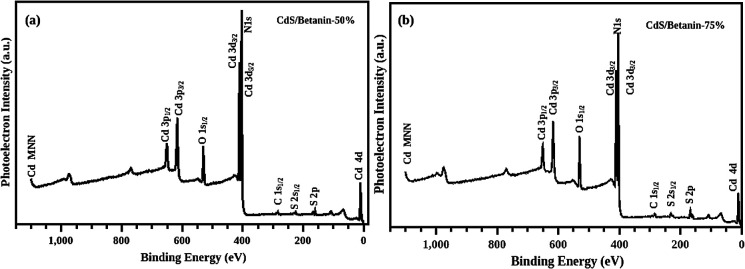
XPS spectra of the sample CdS/betanin −50% (a) and 75% (b) with a depth profiling of 10 nm. The Cd3d peaks is overlapped with N1s at ∼400 eV.

**Fig. 6:**
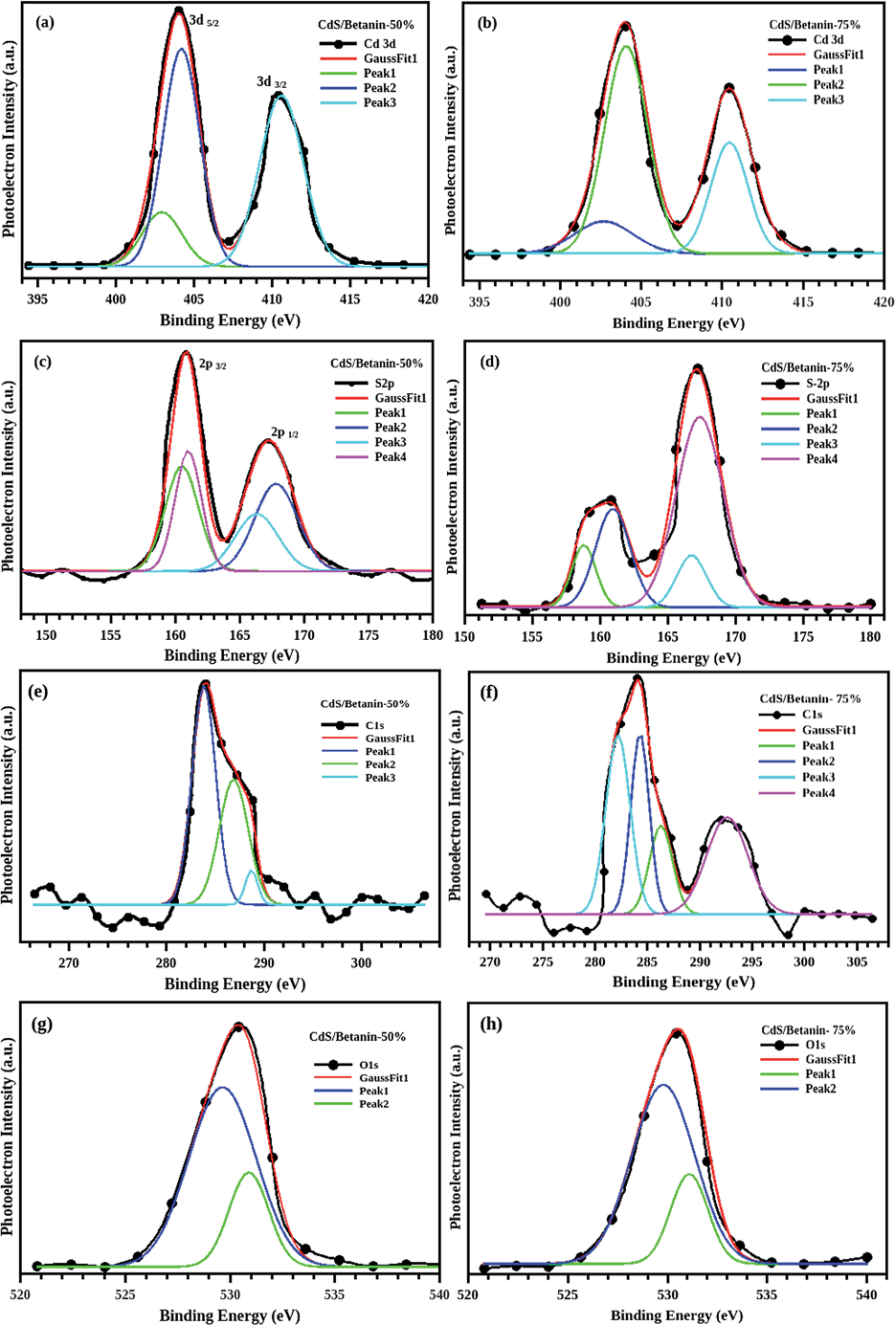
The deconvoluted non Gaussian XPS peaks of Cd3d, S2p, C1s and O1s. The doublet of Cd3d (3d_5/2_ and 3d_3/2_) overlapped with N1s peak at 402 eV for the sample with 50% (a) and 75% (b) betanin. The S2p (c and d) has four deconvoluted peaks, in which peak at161 eV shows the S^2−^ in the composite and all the other peaks above 166 eV shows the oxidized sulphur groups. C–C, C–O, and C=O are the hidden peaks in C1s (e and f). For 75% betanin, instead of peak at 286 eV, a peak at 292 eV is observed indicates –O–C=O– group in the composite. The two peaks of O1s (g and h) clarify the presence of C=O and C–O groups.

The obtained XPS spectra depicted in the Fig. 5 demonstrate the peaks corresponding to the binding energy of the elements including Cd, S, N, C, and O in the composites with different betanin content. The Cd-3d usually has a doublet^26^ and the best fit of the XPS data (Fig. 6(a) and (b)) was obtained with peaks at 404 and 410 eV respectively indicate 3d_5/2_ and 3d_3/2_ due to spin orbit coupling of 3d orbit. In the case of S2p, deconvolution of the non-Gaussian peak (Fig. 6(c) and (d)) predicts four fitted peaks, at ∼160, 161, 166 and 167 eV. The peak at ∼161 eV represents the S^2−^ atom while the peaks above 166 eV assigned as the oxidized peak of sulfur groups found on the surface of the sample.^27–30^ The spectra exhibit well defined C1s and O1s peak which confirm the presence of betanin in the composite. The C1s has three peaks at 284, 286 and 288 eV (Fig. 6(e) and (f)) respectively representing the C–C, C–O, and C=O group. For the sample with 75% betanin content instead of C–O bond at 286 eV, –O–C=O– is observed at 292 eV.^31,32^ The O1s has two peaks, at 529 and 532 eV (Fig. 6(g) and (h)), represents C=O and C–O bonds in the composite. We observed a peak at 402 eV, which is overlapped with the Cd 3d peaks which indicate the presence of =N– in the composite.^33,34^ The peaks at 402 (=N–) 529, 532, 284, 286, 288 and 292 eV show the attachment of betanin in the CdS core. Furthermore, a ratio of 2 : 1 is recorded for N to Cd confirming the attachment of 1 each betanin molecule on a CdS molecule (one betanin molecule possess –NH– and =N– groups).

### Optical studies

#### FT-IR spectroscopy

**Fig. 7:**
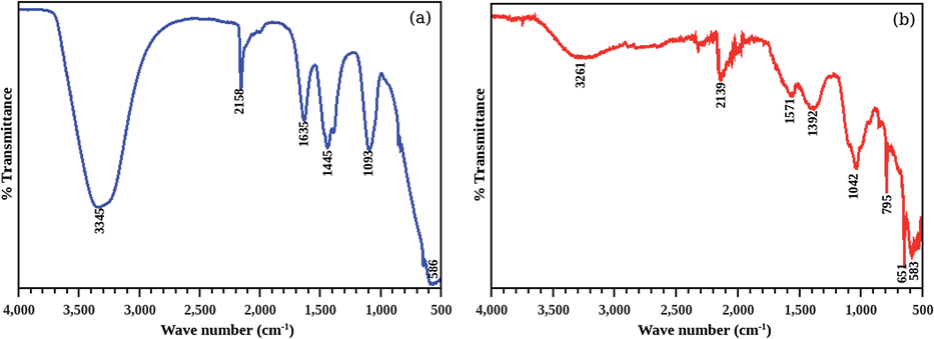
FT-IR spectra of pure CdS (a) and CdS/betanin (b, 50%) nanoparticles. The peaks at 3345, 2158, 1645 cm^−1^ shows the S–OH bond in the CdS nanoparticles and it is shifted to lower energy region to form S–COOH bond in the composite system.

FT-IR spectra of the CdS and CdS/betanin (50%) are recorded in the wavelength range of 4000 to 500 cm^−1^ which are shown in the Fig. 7. The spectrum has two areas^35^ of interest, where the region from 4000 to 1500 cm^−1^ represents the characteristic peak of functional groups known as “important” or “functional group region”. The remaining part (1500 to 400 cm^−1^) is referred as the “finger print region” of the FT-IR spectrum. Here, the FTIR spectra show a broad peak at 3345 cm^−1^ and 2158 cm^−1^ represent the stretching vibration of –OH bond which indicates the strong interaction of CdS with water molecules. The hydroxyl peak in CdS/betanin sample exhibits shift in band position. The presence of hydroxyl group is quite common in the case of CdS grown in solution process,^36^ and the interaction with surroundings takes place *via* hydrogen bonding by the surface bounded H_2_O molecules.^37^ The peak at 1634 cm^−1^ might have originated due to the bending of –OH bond of water molecule.^38^ Since sulfur is treated as a potential H-bond acceptor,^39^ the –OH bond of water is attached to the sulfur atom of CdS. This S…OH bond is replaced by the COOH group of the betanin. The bond length of S…COOH group is comparatively larger and has less energy than S…OH. As a result of this, –OH bonded peaks such as 3345, 2158, 1645 cm^−1^ are shifted towards low energy region (3261, 2139, 1571 respectively) of the FT-IR spectra. This shift in peak confirm the formation CdS/betanin complex structure. Peaks at 1445 and 1093 cm^−1^ represent the trace vibrations of NH^4−^ and SO^4−^ respectively.^40^ The material peaks found in the lower wavelength region, (the peaks 586, 651 and 795 cm^−1^) represent the stretching mode of Cd–S bond. The peaks in the finger print region are also shifted downwards on betanin addition, which may be arose due to the CdS/betanin core/shell formation with thicker shell.

#### UV-Vis spectrophotometry

**Fig. 8:**
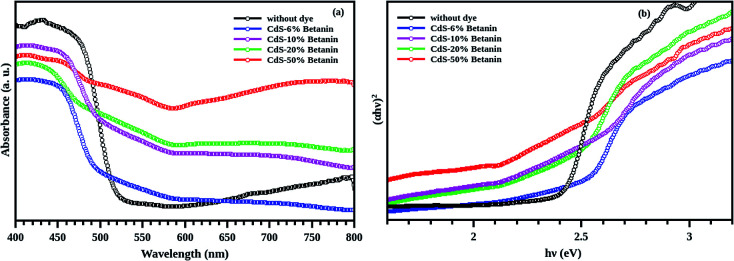
(a) Absorption spectra of CdS with and without betanin dye and (b) the corresponding Tauc plot. The 6% betanin (blue) added sample is blue shifted with respect to the CdS pure, due to the presence of small particles. All the composite has additional peak in the lower wavelength region. The samples 6%, 10% (magenta), 20% (green) and 50% (red) respectively has CdS band edge at 2.46, 2.43, 2.38, 2.16 eV respectively. This shift in energy of the composite hint towards the increased core size with betanin content. The intensity of the peaks increased with the betanin content.

The absorbance of all the samples is recorded in the wavelength range of 400 to 800 nm. The observed results are shown in Fig. 8(a). Effect of betanin on the absorption properties of CdS is clear from the graph. Since CdS is a direct band gap material, nature of transition is direct allowed (ie, *n* = 2), and hence, the *Y*-axis of Tauc plot is (*αhν*)^2^. From the Tauc plot (Fig. 8(b)), the band gap of all the samples are estimated by extrapolating the linear portion to the zero of *X*-axis and the values are tabulated (Table 1).

**Table 1:** Band gap of sample measured from the Tauc plot

Sample	Amount of betanin dye	Band gap of CdS (eV)	Intermediate band
CdS without dye	0%	2.42	—
CdS with betanin dye	6%	2.46	1.90
	10%	2.43	1.89
	20%	2.38	1.88
	50%	2.16	1.62

**Fig. 9:**
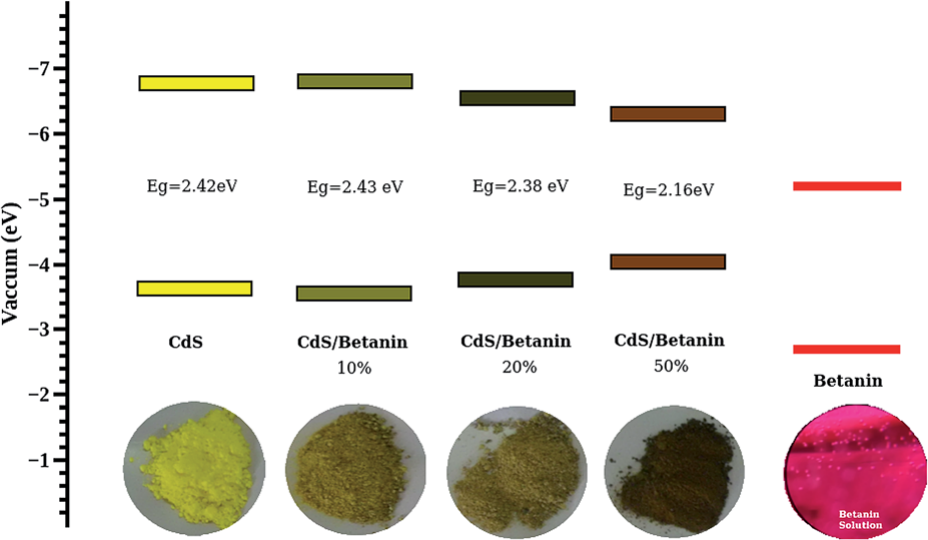
The energy band diagram of CdS and CdS/betanin composite nanoparticles calculated using absolute electronegativity of CdS and the observed band edge of the CdS as well as composites.

**Fig. 10:**
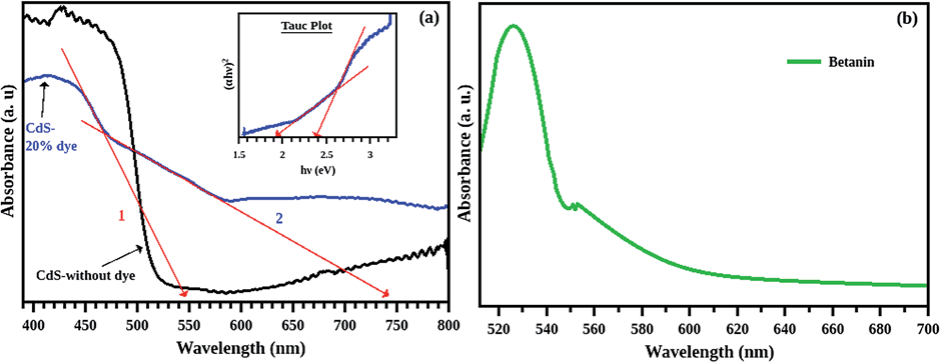
(a) Absorption spectra and Tauc plot (inset) of pure CdS (black) and CdS/betanin composite (blue) with 20% betanin dye (arrow (1)-band edge of CdS, arrow (2)-intermediate band), (b) absorption spectrum of betanin solution which has maximum absorption at 530 nm.

It is observed that the band gaps of CdS/betanin composites are found to be decreased from 2.46 eV to 2.16 eV. The color variation of the sample with dye percentage and the energy band diagram is shown in Fig. 9. The valence (*E*_v_) and conduction (*E*_c_) band edge calculations are done using the relation *E*_v_ = −*χ* − 0.5*E*_g_ and *E*_c_ = –*χ* + 0.5*E*_g_ where −*χ* is the absolute electronegativity (for CdS *χ* = 5.18 (^ref. 41^)). From the band energy diagram, it is clear that the conduction band of betanin^42^ lies in between the conduction and valence band of CdS. The incorporation of dye in CdS has resulted in the formation interband sub levels (Fig. 10(a)) in between VB and CB, thereby reducing the band gap and increasing the tunability of the band edge. Our results support the findings by Okoli *et al.*,^43^ where the band gap of crystalline TiO_2_ (3 eV) was reduced to 2.59 eV on dye sensitization. They used anthocyanin, a natural dye extracted from *Hibiscus sabdariffa* for the sensitization of semiconductor. The shift in absorption observed for the dye sensitized sample may be originated from the complexation of dye molecule with the metal ions.^44^ In most of the cases, impurity addition in CdS reduces the band gap with increase in concentration.^45–47^

The absorption spectra of composite system have an additional band edge (bare CdS has only one absorption edge at 511 nm corresponding to the energy 2.42 eV) in the lower wavelength region. The energy corresponding to the intermediate band (since its value lies below the band gap of the synthesized CdS nanoparticle) is shifted towards the red region with betanin percentage. The intermediate band observed in the energy range of ∼1.75 eV, can help CdS to absorb of lower and higher energy photons simultaneously from the solar radiation, leading to the increased efficiency of CdS based solar cell. Obviously, the intermediate band is resulted from the betanin dye and the behaviour of the absorption spectra hinting towards the formation of core/shell like structure in the composite system with CdS as core and betanin dye as outer shell.

According to Lubedev *et al.*, both theoretical and experimental absorption studies of metal/dye core/shell system has two peaks, one from the metal due the localized surface plasmon resonance and second one due to the electronic excitation of the dye. The peak corresponds to the metal shift towards the higher wavelength region with an increase in shell thickness. Similarly, the absorption edge corresponds to dye also shift towards the red region with shell thickness. Here, the absorption peak of betanin is obtained at ∼530 nm (Fig. 7(b)) and get shifted to a wavelength of ∼650 nm and is higher for the composite system. In account of the theoretical approach of Lubedev *et al.*, we can predict the behaviour of our CdS/betanin core/shell composite system. The intensity of first (CdS) and the second (betanin) absorption edge increases with betanin content which implies that the core CdS nanoparticle has fixed size and the shell thickness is varying with dye percentage. In addition to the shift in absorption edge, the intensity also increased with betanin content. The concentration of the betanin solution is a factor that affects the absorption edge. For less concentrated solution, no additional peak corresponds to betanin was observed (ESI Fig. S1(a)†). As the dye percentage increases from 50% to larger amount, redshift in absorption edge is observed. The band energy corresponds to CdS will decrease further. This will reduce the higher energy photon absorption, and affects the output result. In general, the optical characterisation of the composite nanoparticles clearly demonstrates the importance of geometrical parameters of composite structures in tailoring the optical properties.

### Dielectric studies

**Fig. 11:**
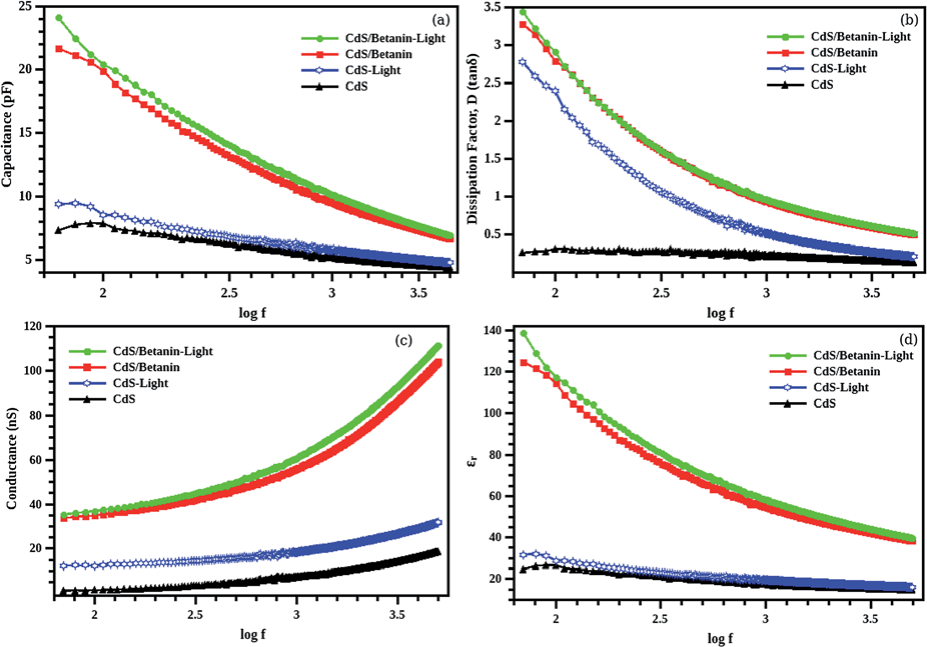
(a) Capacitance (b) dissipation factor (c) conductance and (d) dielectric constant of CdS and CdS/betanin (50%) samples with and without light illumination. Enhanced capacitance, D-factor, conductance and dielectric constant is observed for light illuminated samples. Among CdS (black), illuminated CdS (blue), CdS/betanin composite (red), the illuminated composite (green) system has higher conductance of the order of nano Siemens (nS).

The dielectric studies of the CdS as well as CdS/betanin composite (50% betanin) are shown in Fig. 11. Capacitance (*C*), tangent of the dielectric loss or the dissipation factor (tan *δ*) and conductance (*G*) of the pelletized sample is measured at room temperature. Illumination with 300 W incandescent bulb is done to study the effect of light on the conductivity of the samples at different intensities by changing the distance between the light source and the sample holder (25, 50, 75 cm) as well as the time of illumination. The dielectric constant (*ε*_r_) of the samples is calculated from the equation*ε*_r_ = *Cd*/*ε*_0_*A*where *d* is the thickness and *A* is the area of the sample. From the measured conductance, conductivity (*σ*) is calculated using the following general formula.*σ* = *Gd*/*A*

**Fig. 12:**
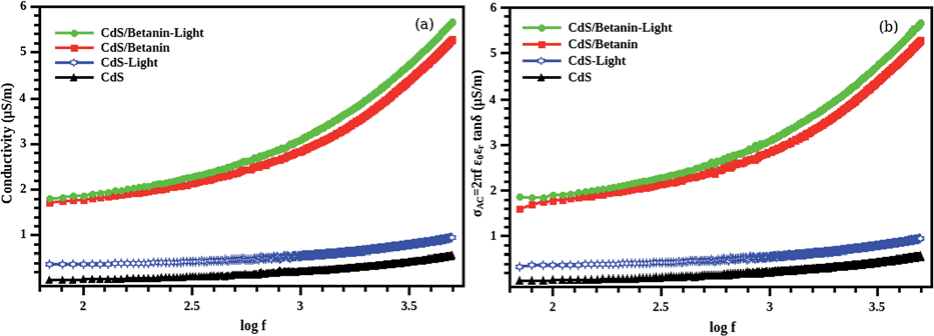
Calculated conductivity of CdS and CdS/betanin composite (50%) structure with two different methods ((a) using conductance (b) using dielectric constant and dissipation factor). Both case enhanced conductance is observed for the light illuminated composite (green) system.

All the quantities are plotted as a function of frequency (50 Hz to 5000 Hz). The properties such as capacitance, dissipation factor and conductance are extremely larger for CdS/betanin core/shell structure. Clearly the modification is resulted from the betanin dye. Almost all the semiconductors show slight increase in conductivity upon light illumination, due to the increase in carrier charge density. Here, the CdS's conductivity (Fig. 12) is increased upon light illumination, since the energy for knocking out the electron from the valence band to the conduction band is made available in the optical form. Concurrently, a huge enhancement in conductivity is observed in CdS/betanin composites on illumination due to the transfer of photogenerated electrons from the conduction band of betanin to the conduction band of CdS.^48^ This will create large number of free carriers in the conduction band of CdS in the composite system resulting in an increased electrical conductivity. Our findings agree with Nabanita Pal *et al.*^49^ They reported an enhanced photoconductivity in mesoporous titania doped with rose bengal dye than the undoped one.

**Fig. 13:**
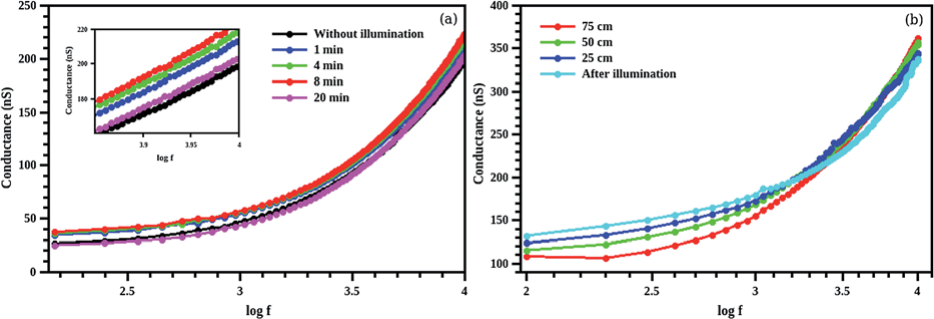
The photo conductance of CdS/betanin composite (50%) at different illumination time (a) and different intensity (b) of light. The inset shows the zoomed view of the conductance. Up to 8 minute the conductance of the sample increased with light illumination, while for 20 min, due to the degradation of the betanin dye, drop in conductance is observed. The intensity of the white light also affects the conductance of the composite by increasing the conductance in the lower frequency region (1 kHz), decreasing in the higher frequency region (10 kHz).

Time of illumination also affects the conductance of the composite structure (Fig. 13(a)). We illuminated the samples for 1, 4, 8 and 20 minutes keeping the sample-light source distance as fixed (25 cm). The maximum conductance is observed for an illumination of 8 minutes. This hike in conductivity is due to the interband transition assisted by the betanin dye. The conductance of the sample is found to be dropped at an illumination of 20 minutes to the conductance value of CdS without illumination. This may be due to the degradation of dye^20^ resulted from the higher intensity light illumination. Sample and light source distance is varied from 25 to 75 cm with a step size of 25 cm (illumination time is fixed less than 1 min). The conductance is found to be increased in the lower frequency (up to 2 kHz) region as the light intensity is increased (25 cm distance has the max light illumination intensity) and is found to be decreased in the higher frequency region (Fig. 13(b)). Higher conductance value is obtained at a distance of 75 cm (min intensity) for higher frequency (10 kHz). After measuring the effect of highest intensity light on the composite sample, the light source is switched off and measured the conductance. Maximum conductance is observed in the low frequency (1 kHz) region and is decreased as the frequency reached to 10 kHz. This decrease may be resulting from the decay process that usually observed in dyes.^50,51^ This phenomenon is known as bleaching effect^52^ in dye molecules due the exposure of highly intense light. As a result of this, the dye molecule may detach from the CdS core and introduce electrons trap centers.^53^ These trap centres at the surface will be attracted by the atmospheric oxygen and cause electron scavenging which will result in a decrease of conductance *via* decreasing the free carrier charge formation in the core/shell system.

## Conclusions

In summary, we have successfully synthesized organic inorganic hybrid core/shell nanostructures by wet chemical route. CdS was the chosen inorganic core, while betanin, an organic dye, was deposited as the shell. The morphological analyses show that the average core diameter of the system was ∼10 nm. It was observed that the deposition of an organic shell of betanin over the CdS core resulted in the modification of the band edge and the band gap was found to be red shifted by 0.26 eV from the pure CdS nanoparticles. The thermal analyses showed that the pure betanin has a disintegration temperature ∼200 °C while the core/shell structures showed high thermal stability, even better than the parent CdS nanoparticles, ruling out the chance of precipitation of betanin separately. To confirm the same, FTIR and XPS analyses were performed. The FTIR spectra showed shift in peaks corresponding to S–OH bond of the CdS nanoparticles to lower energy region to form S–COOH bond in the composite system, while the XPS analysis conducted up to a depth profile of 10 nm showed the attachment of betanin to the CdS with an overall Cd : N ratio as ∼1 : 2. The conductivity and photoconductivity of the CdS/betanin core/shell structures were found much superior to the parent CdS nanoparticles. Hence, from this current investigation, we demonstrate the possibility to employ the inorganic–organic hybrid quantum dots and other nanostructures for enhanced photoconductivity which can offer more efficient thin film energy harvesters.

## Conflicts of interest

There are no conflicts to declare.

## Supplementary Material

RA-008-C7RA13116J-s001
