# Supplementary material for: Enhanced photoconductivity in CdS/betanin composite nanostructures
Source: RSC Adv. 2018 Mar 21;8(21):11330–7. doi: 10.1039/c7ra13116j (PMC9079048; doi:10.1039/c7ra13116j)
Supplement: RA-008-C7RA13116J-s001 [file RA-008-C7RA13116J-s001.pdf]

Electronic Supplementary Material (ESI) for RSC Advances.
This journal is © The Royal Society of Chemistry 2018

SUPPLIMENTARY Figure-S1

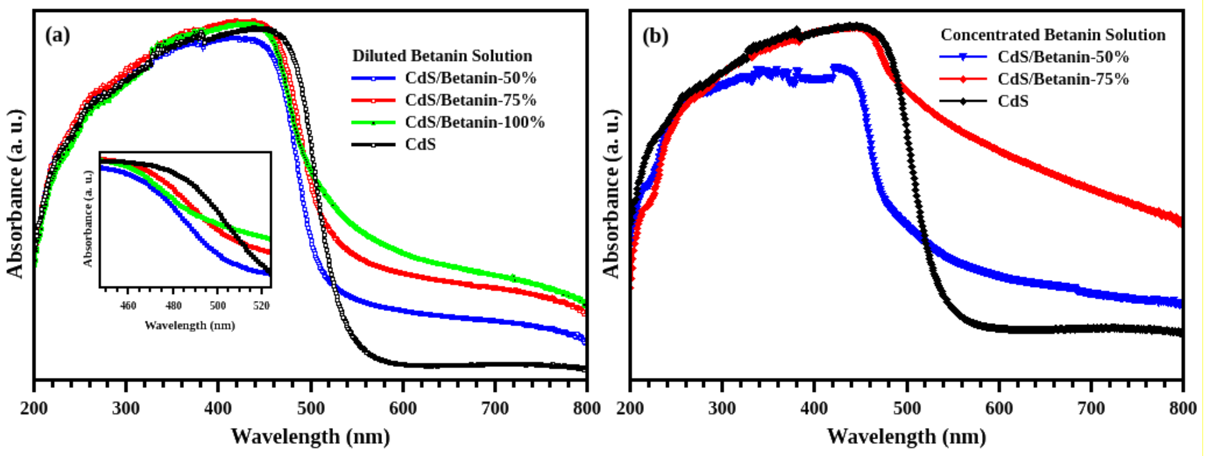

Fig S1: The absorbance of CdS and CdS/Betanin composite at diffrent Betanin concentration. The additional band formation is observed only in samples prepared using concentrated Betanin solution. The optical properties of the composite are purely depending on the concentration of the Betanin solution. Less concentrated Betanin added samples has shift in absorption edge, but has no additional peak observed in the higher wavelength region.
